# Hypoalbuminemia and Hospitalization Risk in Mexican Patients With End-Stage Renal Disease on Hemodialysis: A Single-Center, Retrospective, Cross-Sectional Study

**DOI:** 10.7759/cureus.99560

**Published:** 2025-12-18

**Authors:** David Rojano-Mejía, Juan Figueroa-García, Ruth Ramírez-Fuentevilla, Ernesto Roldan-Valadez, Daniel Martínez-Barro, Juan Carlos H Hernández-Rivera, María Fernanda Figueroa-Hernández, Saúl Eduardo Contreras-Sánchez

**Affiliations:** 1 Health Research Coordination, Instituto Mexicano del Seguro Social, Mexico City, MEX; 2 Family Medicine, Centro de Investigación Educativa y Formación Docente, OOAD Sur de la Ciudad de México, Instituto Mexicano del Seguro Social, Mexico City, MEX; 3 Coordination of Planning and Institutional Liaison, Instituto Mexicano del Seguro Social, Mexico City, MEX; 4 Division of Research, Instituto Nacional de Rehabilitacion "Luis Guillermo Ibarra Ibarra" (INR), Mexico City, MEX; 5 Department of Radiology, I.M. Sechenov First Moscow State Medical University (Sechenov University), Moscow, RUS; 6 Physical Medicine and Rehabilitation, Hospital General Regional N. 6, OOAD Tamaulipas, Instituto Mexicano del Seguro Social, Ciudad Madero, MEX; 7 Nephrology, Unidad de Investigación Médica en Enfermedades Nefrológicas, Hospital de Especialidades, Centro Médico Nacional Siglo XXI, Instituto Mexicano del Seguro Social, Mexico City, MEX; 8 Internal Medicine, Hospital General de Zona 47, OOAD Sur del D. F. Instituto Mexicano del Seguro Social, Mexico City, MEX; 9 Epidemiology and Health Services Research Unit CMN Siglo XXI, Instituto Mexicano del Seguro Social, Mexico City, MEX

**Keywords:** chronic kidney disease, end-stage renal disease, hemodialysis, hospitalization, hypoalbuminemia, serum albumin

## Abstract

Background and objective

Hypoalbuminemia in hemodialysis (HD), commonly defined as serum albumin <3.5 g/dL, is multifactorial and linked to adverse outcomes, including higher hospitalization rates. We aimed to evaluate the association between hypoalbuminemia and hospitalization among adults with end-stage renal disease (ESRD) on maintenance HD at a secondary-care hospital in Mexico.

Methods

We performed a retrospective, cross-sectional, analytical study of clinical records from July 2022 to June 2023. Patients were categorized into two groups: Group 1 (G1), those with hypoalbuminemia (<3.5 g/dL), and Group 2 (G2), those with normoalbuminemia (≥3.5 g/dL). The primary outcome was hospitalization (yes/no), with length of stay as a secondary outcome. Comparisons between groups were performed using the Chi-square test for categorical variables and the Mann-Whitney U test for continuous variables with non-normal distributions. Odds ratios (ORs) with 95% confidence intervals (95% CI) were estimated, and a two-sided p value <0.05 was considered statistically significant.

Results

We analyzed a total of 226 records (mean age: 49.8 ± 16.2 years; 60.6% male) (mean age: 49.8 ± 16.2 years; 60.6% male), with diabetes being the most common comorbidity. Hospitalization was observed in 77.0% of patients in G1 compared with 25.7% in G2, demonstrating a strong association (χ² = 59.58, p<0.001) and a crude OR of 9.69 (95% CI: 5.60-16.50). The length of hospital stay was significantly longer in G1 than in G2 (11.82 ± 12.69 vs. 3.96 ± 9.40 days; p<0.001).

Conclusions

Hypoalbuminemia in HD patients is strongly associated with both higher hospitalization risk and longer hospital stays. Routine albumin surveillance may help identify high-risk patients and prompt targeted interventions - nutritional optimization, infection/inflammation management, and dialysis adequacy checks - to reduce healthcare utilization and improve clinical outcomes.

## Introduction

Chronic kidney disease (CKD) is a major public health challenge worldwide and in Mexico [1]. In 2021, its global prevalence was estimated at 673.7 million cases [2]. In Mexico, about 11% of the population is affected (largely driven by chronic degenerative diseases), placing a substantial financial burden on public health institutions such as the Instituto Mexicano del Seguro Social (IMSS), which cares for most patients requiring dialysis or transplantation [1]. According to international guidelines (Kidney Disease: Improving Global Outcomes (KDIGO), CKD is defined by a sustained reduction in glomerular filtration rate (GFR <60 mL/min/1.73 m²) and/or markers of kidney damage for at least three months. Patients who progress to end-stage renal disease (ESRD; CKD G5) require renal replacement therapy (RRT) to survive [3-5]. In Mexico, hemodialysis (HD) is a common RRT modality but is frequently complicated by protein-energy wasting and inflammation [6].

Serum albumin, the most abundant plasma protein, maintains oncotic pressure and transports endogenous and exogenous molecules [7,8]. Hypoalbuminemia, typically defined as serum albumin ≤3.5 g/dL, is common at dialysis initiation and is a strong predictor of hospitalization and mortality [8]. In HD patients, hypoalbuminemia has multifactorial causes, including reduced hepatic synthesis (malnutrition), increased catabolism and inflammatory activity, and dialysis-related amino-acid/albumin losses. Although lower albumin has been linked to longer hospital stays, data from Mexican HD populations remain limited [9]. Monitoring albumin thus provides a pragmatic, inexpensive way to identify high-risk patients and to guide timely nutritional and anti-inflammatory interventions [10-12]. In this study, we aimed to evaluate the association between hypoalbuminemia and hospitalization risk in Mexican patients with ESRD undergoing HD at a secondary-care hospital in the metropolitan area of central Mexico.

## Materials and methods

Study design and setting

We conducted a retrospective, cross-sectional, analytical study of adults with ESRD receiving maintenance HD at a secondary-care hospital in the metropolitan area of central Mexico. Following institutional approval, data were retrospectively extracted from the clinical records of patients who underwent HD between July 2022 and June 2023.

Participants (eligibility criteria)

Inclusion Criteria

Eligible records included those of patients aged 18 years or older, of either sex, with ESRD undergoing HD during the study period, who had a documented serum albumin measurement within the predefined baseline window and a complete medical record in accordance with the Mexican Official Standard NOM-004-SSA3-2012 (clinical record).

Exclusion Criteria

We excluded records of patients aged >85 years, those with chronic liver disease (Child-Pugh classes A-C), advanced heart failure, or active malignancy receiving chemotherapy; records with key missing data for primary variables were also excluded.

Exposure and grouping

Serum albumin (g/dL) was recorded from the clinical record. Patients were classified a priori into two groups: Group 1 (G1) - hypoalbuminemia (<3.5 g/dL) and Group 2 (G2) - normoalbuminemia (≥3.5 g/dL).

Outcomes and variables

The primary outcome was hospitalization (yes/no) during follow-up in the study window. Secondary outcomes included length of hospital stay (days) and cause of hospitalization. Additional covariates comprised sociodemographic factors (age, sex, education level, and marital status, including single, married, widowed, and cohabiting union), as well as major comorbidities systematically documented, namely diabetes mellitus and hypertension, which are the leading causes of ESRD in our setting, dialysis vintage, and baseline laboratory parameters. Other etiologies and comorbid conditions (such as glomerulonephritis, autosomal dominant polycystic kidney disease, tubulointerstitial nephritis, and alcohol-related liver disease) were not consistently documented across clinical records and therefore could not be characterized in detail.

Sample size

An a priori sample-size estimation for comparing two proportions (hypoalbuminemia vs. normoalbuminemia) was performed assuming a two-sided α of 0.05, 80% power, and a 1:1 allocation ratio. Expected proportions for the primary outcome (hospitalization) were derived from the literature, with rates of 38% in G1 and 21% in G2 [13]. Based on these assumptions, a total sample of 226 patients (113 per group) was required, corresponding to an approximate odds ratio (OR) of 2.3, a relative risk (RR) of 1.8, and an absolute risk difference of about 17%. The final cohort included 226 eligible records, thereby achieving the prespecified sample size.

Statistical analysis

Data were entered into a spreadsheet and analyzed using IBM SPSS Statistics (IBM Corp., Armonk, NY). Continuous variables were summarized as mean (standard deviation (SD) or median (interquartile range (IQR), aas appropriate, while categorical variables were expressed as counts and percentages. Group comparisons used the Chi-square test (or Fisher’s exact test when expected counts were <5) for categorical variables and t-test or Mann-Whitney U test for continuous variables, according to distributional assumptions. Effect sizes for categorical comparisons were reported as φ (2×2) or Cramer’s V (>2×2). The association between albumin category and hospitalization was expressed as ORs with 95% confidence intervals (CIs). A two-sided p-value <0.05 was considered statistically significant.

Ethical considerations

The protocol (registration R-2022-1402-037) was approved by the institutional research and ethics committee on September 22, 2022. The study complied with applicable national regulations on health research and personal data protection.

## Results

Baseline characteristics

Among 226 patients, 113 had hypoalbuminemia (<3.5 g/dL) and 113 had normoalbuminemia (≥3.5 g/dL). Patients in the hypoalbuminemia group were older (mean age: 53.1 ± 16.2 vs. 46.4 ± 15.5 years; p = 0.002). Sex distribution, education level, and marital status did not differ significantly between groups (Table 1).

**Table 1:** Sociodemographic characteristics of the study population by serum albumin group G1 = hypoalbuminemia (<3.5 g/dL); G2 = normoalbuminemia (≥3.5 g/dL). P-values compare G1 vs. G2 (t-test for the continuous variable (Age); Chi-square test for categorical variables; Fisher’s exact test used when expected cell counts <5). Percentages are column percentages; totals may not equal 100% due to rounding. A p-value <0.05 was considered statistically significant SD: standard deviation; NA: not applicable

Characteristic	G1 (n = 113)	G2 (n = 113)	Total (n = 226)	P-value
Age, years, mean ± SD	53.1 ± 16.2	46.4 ± 15.5	49.8 ± 16.2	0.002
Sex, n (%)				0.135
Male	63 (55.8)	74 (65.5)	137 (60.6)	NA
Female	50 (44.2)	39 (34.5)	89 (39.4)	NA
Education, n (%)				0.273
Primary	28 (24.8)	24 (21.2)	52 (23.0)	NA
Secondary	34 (30.1)	26 (23.0)	60 (26.5)	NA
High school	32 (28.3)	37 (32.7)	69 (30.5)	NA
Bachelor’s	11 (9.7)	22 (19.5)	33 (14.6)	NA
No formal education	8 (7.1)	4 (3.5)	12 (5.3)	NA
Marital status, n (%)				0.326
Married	69 (61.1)	70 (61.9)	139 (61.5)	NA
Single	30 (26.5)	33 (29.2)	63 (27.9)	NA
Cohabiting union	5 (4.4)	8 (7.1)	13 (5.8)	NA
Widowed	9 (8.0)	2 (1.8)	11 (4.9)	NA

Comorbidities and clinical characteristics

Diabetes mellitus was more frequent in the hypoalbuminemia group (60.2% vs. 38.9%; p = 0.001; φ = 0.21). Hypertension was markedly more frequent in the normoalbuminemia group than in the hypoalbuminemia group (92.0% vs. 39.8%; p = 0.001; φ = 0.55). BMI category distribution was not statistically different overall (p = 0.147), although morbid obesity occurred more often with hypoalbuminemia (6.2% vs. 0.9%). Patients with hypoalbuminemia tended to be newer to HD (≤1 year: 74.3% vs. 62.8%; p = 0.091) (Table 2).

**Table 2:** Comorbidities and clinical characteristics by serum albumin group G1 = hypoalbuminemia (<3.5 g/dL); G2 = normoalbuminemia (≥3.5 g/dL). P-values compare distributions between G1 and G2 using Chi-square tests (Fisher’s exact test applied when any expected cell count <5). Effect sizes are reported as φ for 2×2 tables and Cramer’s V for tables with more than two categories. Percentages are column percentages; totals may not equal 100% due to rounding. A p-value <0.05 was considered statistically significant NA: not applicable

Characteristic	G1 (n = 113)	G2 (n = 113)	Total (n = 226)	P-value	Effect size
Diabetes, n (%)	68 (60.2)	44 (38.9)	112 (49.6)	0.001	φ = 0.21
Hypertension, n (%)	45 (39.8)	104 (92.0)	149 (65.9)	0.001	φ = 0.55
Body mass index, n (%)				0.147	Cramer’s V = 0.16
Normal	64 (56.6)	71 (62.8)	135 (59.7)	NA	NA
Overweight	28 (24.8)	28 (24.8)	56 (24.8)	NA	NA
Obesity class I	10 (8.8)	10 (8.8)	20 (8.8)	NA	NA
Obesity class II	4 (3.5)	3 (2.7)	7 (3.1)	NA	NA
Morbid obesity	7 (6.2)	1 (0.9)	8 (3.5)	NA	NA
Dialysis duration, years, n (%)				0.091	Cramer’s V = 0.18
≤1	84 (74.3)	71 (62.8)	155 (68.6)	NA	NA
1–5	24 (21.2)	36 (31.9)	60 (26.5)	NA	NA
5.1–10	5 (4.4)	5 (4.4)	10 (4.4)	NA	NA
≥10	0 (0)	1 (0.9)	1 (0.4)	NA	NA

Hospitalization outcomes

Hospitalization occurred in 87/113 (77.0%) with hypoalbuminemia vs. 29/113 (25.7%) with normoalbuminemia, yielding an OR of 9.69 with a highly significant association (χ² = 59.58, p<0.001). Mean length of stay was longer with hypoalbuminemia (11.82 ± 12.69 vs. 3.96 ± 9.40 days; p<0.001). For clinical framing, the absolute risk difference was 51.3 percentage points, and the relative risk was ~3.0 (Table 3).

**Table 3:** Association between hypoalbuminemia and hospitalization. G1 = hypoalbuminemia (<3.5 g/dL); G2 = normoalbuminemia (≥3.5 g/dL). For the hospitalization comparison, the p-value is from a Chi-square test, and the OR with 95% CI is the crude estimate derived from the 2×2 table. For length of stay, groups were compared using the Mann–Whitney U test. All tests are two-sided with α = 0.05. A p-value <0.05 was considered statistically significant OR: odds ratio; CI: confidence interaval; SD: standard deviation; NA: not applicable

Outcome	G1 (n = 113)	G2 (n = 113)	OR (95% CI)	P-value
Hospitalization, n/N (%)	87/113 (77.0)	29/113 (25.7)	9.69 (5.60–16.50)	<0.001
Hospital stay, days, mean ± SD	11.82 ± 12.69	3.96 ± 9.40	NA	<0.001

## Discussion

Principal findings

In this single-center cohort of adults on maintenance HD, hypoalbuminemia (<3.5 g/dL) was strongly associated with hospitalization. Patients with low albumin had substantially higher admission rates (77.0% vs. 25.7%) and longer mean length of stay (11.8 vs. 4.0 days), yielding a large crude effect size (OR: 9.69; 95% CI: 5.60-16.50). These findings reinforce the clinical usefulness of serum albumin as an easily obtainable marker that integrates inflammation, protein-energy status, and overall illness severity in ESRD [14-16]

Comparison with prior literature

Our findings are consistent with contemporary HD cohorts in which lower baseline albumin has been associated with higher hospitalization rates and longer length of stay, even after multivariable adjustment [16]. Similar to these studies, we observed a markedly higher proportion of patients with at least one admission and more hospital days among those with hypoalbuminemia. In line with recent KDIGO and Kidney Disease Outcomes Quality Initiative (KDOQI) guidelines, our results support the use of serum albumin as a pragmatic prognostic marker within a broader, multidimensional assessment of nutritional and inflammatory status in ESRD [3,17].

Plausible biological mechanisms

Several mechanisms plausibly link hypoalbuminemia to hospitalization risk. Inflammation suppresses hepatic albumin synthesis and increases catabolism; dialytic and gastrointestinal losses further reduce circulating levels; reduced intake and intercurrent illness compound these effects [18,19]. Within this context, albumin functions as a summary signal of the malnutrition-inflammation complex and the broader protein-energy wasting construct - both recognized drivers of adverse outcomes in HD [20,21]. The longer stays observed among patients with low albumin in our study fit this mechanistic framework [22,23].

Clinical implications

Routine, standardized surveillance of serum albumin can help identify patients at elevated risk of admission and prolonged stays and can trigger bundled, multidisciplinary actions: dietetic assessment and individualized protein/energy prescriptions; active management of infection and inflammation; optimization of dialysis adequacy; and consideration of oral or intradialytic supplementation when appropriate [17]. In health systems with resource constraints and discontinuities of care, a simple marker like albumin can support triage, prioritization for closer follow-up, and earlier intervention [9].

Importantly, recent evidence suggests that the prognostic value of albumin may be modified by patient age. A prospective study found hypoalbuminemia to be more strongly predictive of mortality in HD patients under 65 years compared to older individuals, recommending age‐adjusted cut-offs (3.7 g/dL for <65 years vs. 3.5 g/dL for ≥65 years) to improve risk stratification [23]. Although our current analysis did not employ stratification by age, future work should consider age-specific albumin thresholds to refine clinical interventions. Additionally, the link between albumin levels and renal prognosis in earlier stages of CKD has been highlighted in cohort data. For example, patients with serum albumin below 4.1 g/dL demonstrated markedly worse renal outcomes and faster eGFR decline, underscoring albumin’s role even before dialysis initiation [24].

Analysis within our cohort

Baseline characteristics were broadly comparable between groups, although diabetes was more frequent with hypoalbuminemia, BMI distributions were similar overall, and morbid obesity was uncommon but somewhat more prevalent in the hypoalbuminemia group. Additionally, patients with low albumin tended to be at an earlier stage of their dialysis treatment. Together, these patterns describe a phenotype of metabolic and inflammatory vulnerability that may require proactive surveillance and targeted support. The absolute risk difference in hospitalization (about 51 percentage points) highlights a clinically meaningful gradient relevant to bed capacity, cost, and patient experience.

Collectively, our findings reinforce the critical importance of albumin as a prognostic biomarker in ESRD undergoing dialysis, particularly in the Mexican context, where baseline data had been scarce. A key strength of our study lies in its focus on a Mexican population, representing a meaningful contribution to the existing scientific literature. Research addressing hypoalbuminemia in ESRD patients undergoing HD within the Latin American context remains limited. Our findings, therefore, provide valuable region-specific data and complement evidence that has thus far been predominantly derived from non-Latin American cohorts. From a clinical perspective, interventions aimed at ameliorating hypoalbuminemia, such as optimizing nutritional support, inflammatory control, and appropriate selection of dialysis membranes, are highly warranted. Moreover, implementing routine albumin monitoring in conjunction with comprehensive nutritional assessments can facilitate the timely identification of at-risk patients and targeted preventive strategies.

Strengths and limitations

Key strengths of this study include a prespecified albumin threshold, complete case capture in a defined window, and clear, easily interpretable outcomes. However, the retrospective, single-center design limits causal inference and generalizability. Residual confounding by comorbidity burden, inflammatory status, dialysis adequacy, or socioeconomic factors is possible. As this was a retrospective review of clinical records, detailed and standardized information on some etiologies and comorbid conditions (e.g., glomerulonephritis, autosomal dominant polycystic kidney disease, tubulointerstitial nephritis, and alcohol use disorder or alcohol-related liver disease) was not consistently available in the clinical records, preventing full analysis and leaving the possibility of residual confounding. We reported crude associations; if multivariable modeling is pursued, essential covariates should include age, sex, diabetes, dialysis vintage, and a comorbidity summary, with adjusted ORs and CIs reported explicitly. Cause-specific admissions were not analyzed; stratifying by infection, cardiovascular events, access complications, and other drivers would sharpen mechanistic inference and guide targeted interventions.

Implications for practice and research

Dialysis programs could integrate albumin-based risk flags into routine patient reviews to trigger bundled actions and structured follow-up. Prospective studies should evaluate whether such bundles reduce admissions and shorten stays among patients with persistently low albumin. Additionally, future research could incorporate inflammatory biomarkers and functional measures to improve risk stratification beyond albumin alone [3].

## Conclusions

In this single-center cohort of adults receiving maintenance HD, hypoalbuminemia (<3.5 g/dL) was strongly associated with greater healthcare utilization: patients with low albumin had a substantially higher probability of hospitalization and longer mean hospital stays. Although these are crude, observational estimates potentially affected by residual confounding, they underscore the value of serum albumin as a low-cost, reproducible risk indicator that integrates illness severity, inflammation, and protein-energy status. From a clinical perspective, routine albumin surveillance can be used to trigger a bundled response - nutrition assessment and individualized protein/energy prescriptions, active evaluation and treatment of infection and inflammation, optimization of dialysis adequacy, and early follow-up for those below threshold. Programs operating under resource constraints may benefit from incorporating an albumin alert into routine dialysis reviews to help prioritize high-risk patients. Future studies should validate these findings in multicenter cohorts, use multivariable adjustment for case mix and dialysis vintage, and analyze cause-specific hospitalizations to identify the most actionable pathways. Nonetheless, our data support incorporating albumin-based risk stratification into routine HD care to help reduce admissions and shorten length of stay.

## References

[1] Méndez-Durán A (2021). Evolution of renal replacement therapy in Mexico in the last 10 years. Nefrologia (Engl Ed).

[2] Guo J, Jiao W, Xia S, Xiang X, Zhang Y, Ge X, Sun Q (2025). The global, regional, and national patterns of change in the burden of chronic kidney disease from 1990 to 2021. BMC Nephrol.

[3] (2024). KDIGO 2024 clinical practice guideline for the evaluation and management of chronic kidney disease. Kidney Int.

[4] Maringhini S, Zoccali C (2024). Chronic kidney disease progression-a challenge. Biomedicines.

[5] Murton M, Goff-Leggett D, Bobrowska A (2021). Burden of chronic kidney disease by KDIGO categories of glomerular filtration rate and albuminuria: a systematic review. Adv Ther.

[6] Argaiz ER, Morales-Juárez L, Razo C (2021). The burden of chronic kidney disease in Mexico. Data analysis based on the Global Burden of Chronic Kidney Disease study. Disease.

[7] Wang X, Chu H, Zhou H (2023). Association between hypoalbuminemia and mortality in patients undergoing continuous renal replacement therapy: a systematic review and meta-analysis. PLoS One.

[8] Manolis AA, Manolis TA, Melita H, Mikhailidis DP, Manolis AS (2022). Low serum albumin: a neglected predictor in patients with cardiovascular disease. Eur J Intern Med.

[9] Vasquez-Jimenez E, Madero M (2020). Global dialysis perspective: Mexico. Kidney360.

[10] Brookes EM, Power DA (2022). Elevated serum urea-to-creatinine ratio is associated with adverse inpatient clinical outcomes in non-end stage chronic kidney disease. Sci Rep.

[11] Bansal N, Zelnick L, Bhat Z (2019). Burden and outcomes of heart failure hospitalizations in adults with chronic kidney disease. J Am Coll Cardiol.

[12] Bargetzi A, Emmenegger N, Wildisen S (2021). Admission kidney function is a strong predictor for the response to nutritional support in patients at nutritional risk. Clin Nutr.

[13] Antunes SA, Canziani ME, Campos AF, Vilela RQ (2016). Hypoalbuminemia seems to be associated with a higher rate of hospitalization in hemodialysis patients. J Bras Nefrol.

[14] Song H, Wei C, Hu H, Wan Q (2022). Association of the serum albumin level with prognosis in chronic kidney disease patients. Int Urol Nephrol.

[15] Boz G, Uludag K (2021). Serum albumin trends in relation with prognosis of individuals receiving hemodialysis therapy. Cureus.

[16] Uludag K, Boz G, Gunal AI (2021). Lower serum albumin level is associated with increased risk of hospital admission and length of stay in hospital among incident hemodialysis patients by using overdispersed model. Ther Apher Dial.

[17] Ikizler TA, Burrowes JD, Byham-Gray LD (2020). KDOQI clinical practice guideline for nutrition in CKD: 2020 update. Am J Kidney Dis.

[18] Kalantar-Zadeh K, Ficociello LH, Bazzanella J, Mullon C, Anger MS (2021). Slipping through the pores: hypoalbuminemia and albumin loss during hemodialysis. Int J Nephrol Renovasc Dis.

[19] Wang CH, Jiang MH, Ma JM (2024). Identification of independent risk factors for hypoalbuminemia in patients with CKD stages 3 and 4: the construction of a nomogram. Front Nutr.

[20] Kalantar-Zadeh K, Ikizler TA, Block G, Avram MM, Kopple JD (2003). Malnutrition-inflammation complex syndrome in dialysis patients: causes and consequences. Am J Kidney Dis.

[21] Fouque D, Kalantar-Zadeh K, Kopple J (2008). A proposed nomenclature and diagnostic criteria for protein-energy wasting in acute and chronic kidney disease. Kidney Int.

[22] Hamroun A, Frimat L, Laville M (2022). New insights into acute-on-chronic kidney disease in nephrology patients: the CKD-REIN study. Nephrol Dial Transplant.

[23] Barril G, Nogueira A, Alvarez-García G, Núñez A, Sánchez-González C, Ruperto M (2022). Nutritional predictors of mortality after 10 years of follow-up in patients with chronic kidney disease at a multidisciplinary unit of advanced chronic kidney disease. Nutrients.

[24] Cheng T, Wang X, Han Y, Hao J, Hu H, Hao L (2023). The level of serum albumin is associated with renal prognosis and renal function decline in patients with chronic kidney disease. BMC Nephrol.

